# Co-administration of Grape Seed Extract and Exercise Training Improves Endothelial Dysfunction of Coronary Vascular Bed of STZ-Induced Diabetic Rats

**DOI:** 10.5812/ircmj.7624

**Published:** 2013-10-05

**Authors:** Mohammad Badavi, Hassan Ali Abedi, Ali Reza Sarkaki, Mahin Dianat

**Affiliations:** 1 Physiology Research Center, Physiology Department, Faculty of Medicine, Ahvaz Jundishapur University of Medical Sciences, Ahvaz, IR Iran; 2 Physiology Department, Faculty of Medicine, Jahrom University of Medical Sciences, Jahrom, IR Iran

**Keywords:** Diabetic Vascular Complication, Grape Seed Extract, Exercise, Coronary Vessels

## Abstract

**Background:**

One of the known complications of diabetes mellitus is vascular dysfunction. Inability of the coronary vascular response to cardiac hyperactivity might cause a higher incidence of ischemic heart disease in diabetic subjects. It has been indicated that regular exercise training and antioxidants could prevent diabetic cardiovascular problems enhanced by vascular damage.

**Objectives:**

The aim of this study was to determine the effects of grape seed extract (as antioxidant), with and without exercise training on coronary vascular function in streptozotocin induced diabetic rats.

**Materials and Methods:**

Fifty male Wistar rats weighing 200 – 232 grams were randomly divided into five groups of 10 rats each: sedentary control, sedentary diabetic, trained diabetic, grape seed extract (200 mg/kg) treated sedentary diabetic and, grape seed extract treated trained diabetic. Diabetes was induced by one intraperitoneal injection of streptozotocin. After eight weeks, coronary vascular responses to vasoactive agents were determined.

**Results:**

The endothelium dependent vasorelaxation to acetylcholine was reduced significantly in diabetic animals; exercise training or grape seed extract administration partially improves this response. However, exercise training in combination with grape seed extract restores endothelial function completely. The endothelium independent vasorelaxation to sodium nitroprusside was improved by combination of exercise training and grape seed extract. On the other hand, the basal perfusion pressure and vasoconstrictive response to phenylephrine did not change significantly.

**Conclusions:**

The data indicated that co-administration of grape seed extract and exercise training had more significant effects than exercise training or grape seed extract alone; this may constitute a convenient and inexpensive therapeutic approach to diabetic vascular complications.

## 1. Background

Coronary artery disease is a major factor of mortality and morbidity in diabetic patients. Actually, risk of myocardial infarction in diabetics equals that of non-diabetic patients with a history of infarction (1). One of the known complications of diabetes mellitus (DM) is vascular dysfunction (2-4). Inability of the coronary vascular response to cardiac hyperactivity might cause the higher incidence of ischemic heart disease in diabetic subjects (5). Exercise training (ET) has beneficial effects on diabetes and its complications, such as decrease in systemic vascular resistance, heart rate (6), and blood pressure, increase in insulin sensitivity and improvement in blood lipid profile (7). Angiogenesis and change in vascular reactivity was reported in different vascular beds by ET (7). It was shown that regular ET ameliorates endothelial dysfunction through modification of oxidative stress (8).

Oxidative stress has a pivotal role in the pathogenesis of vascular complications of DM (9-11). Thus, improvement of oxidative stress may ameliorate vascular dysfunction induced by DM. Treatment of diabetic rats by antioxidants such as alpha-lipoic acid (9) and high-dose allopurinol (10) reversed endothelial dysfunction induced by streptozotocin (STZ).

The compound of grape seed extract (GSE) has antioxidant properties (12) with greater potency than vitamin C and E (13). It was reported that impaired endothelium dependent relaxation of aortic rings induced by STZ was improved by grape seed proanthocyanidin extract (14). To our knowledge, no study to date has examined effects of GSE on coronary vascular bed response to vasoactive agents. On the other hand, we hypothesized that GSE has potential effects on vascular function to scavenge free radicals and potentiate positive effects of ET.

## 2. Objectives

Because coronary artery disease frequently occurs in diabetic states, therefore, the present study was designed to determine effects of GSE with and without exercise on coronary vascular bed responses of diabetic rats to phenylephrine (PE), sodium nitroprusside (SNP) and acetylcholine (Ach).

## 3. Materials and Methods

### 3.1. Animals

Fifty male Wistar strain rats weighing 200 – 232 grams from the Animal House of the Physiology Research Center at the Ahvaz Jundishapur University of Medical Sciences, Iran, at the beginning of the study were randomly divided in to five groups of 10 rats each: sedentary control (SC), sedentary diabetic (SD), trained diabetic (TrD), GSE treated sedentary diabetic (ExD), and GSE treated trained diabetic (TrExD). Diabetes was induced by an intraperitoneal injection of STZ (60mg/kg body weight) dissolved in 0.3ml of normal saline. Control animals were injected with an equivalent volume of vehicle. Dose of GSE was 200 mg/kg and it was administered orally via gavage, once a day. ET was conducted on a treadmill. Duration of the protocol was 8 weeks. All groups were maintained under the same conditions (temperature-controlled room, 22 °C) with a 12-h dark-light cycle, supplied with food and water ad- libitum. Rats were considered diabetic when blood glucose levels were > 300 mg/dl five days later (15). The experimental protocol and procedures were submitted and approved by the Institutional Animal Care and Use Committee of the University based on the guidelines for care and use of laboratory animals published by the US National Institutes of Health (NIH Publication, revised 1996).

### 3.2. Exercise Training Protocol

As shown in Table 1, rats conducted ET on a treadmill daily for 8 weeks, 1 day after diabetic verification after gavage of GSE. 

**Table 1.** Exercise Training Protocol for Rats on Treadmill

Week	Belt Speed (m/min)	Inclination, Angle	Total Time (min)
**1**	16	0	30
**2**	16	5	30
**3**	16	10	45
**4**	16	12	45
**5**	16	12	60
**6**	16	12	60
**7**	16	12	60
**8**	16	12	60

### 3.3. Preparation of Grape Seed Extract

Vitis Vinifera was confirmed by the Ghazvin Agricultural Research Center, Ghazvin, Iran. Voucher specimen was available in the herbarium at the Department of Phamacognosy, Faculty of Pharmacy, Joundishapur Medical Sciences University, Ahvaz, Iran. Separation of grape seeds from grapes was done manually, dried at room temperature for 7 days and milled to fine powder. The powders were macerated in 70 % ethanol (25% w/v) for 3 days in shade (25 - 30°C) and were stirred 3 times a day. After filtration of the mixture with cheese cloth, the filtrate was dried at room temperature to remove ethanol. Finally grape seed extract was obtained as a powder (16).

### 3.4. Preparation of the Perfused Heart

The animals were anesthetized by sodium pentothal (80 mg/kg, i.p) containing 1000 U/kg heparin. Next, the chest was opened and the heart was rapidly excised and placed into a Petri dish containing ice-cold oxygenated modified Krebs-Henseleit solution (KHS). After washing with ice-cold KHS and arresting, the hearts were cannulated via the ascending aorta and immediately transferred to the Langendorff system. The aorta was retroperfused with a modified KHS of the following composition (mM): NaCl 118.4, KCl 4.7, MgSO_4_ H_2_O 1.2, KH_2_PO_4_ 2H_2_O 1.2, NaHCO_3_ 25, CaCl_2_ 2.5 and glucose 11.1 in distilled water. This solution was maintained at 37 °C, bubbled with 5% CO_2_ and 95% O_2_ and perfused the isolated hearts at a constant rate (4ml/min) via a peristaltic pump (Gilson-France). Maximum time between the excision of the heart and the beginning of perfusion was 2 minutes. After the stabilization period (25-30 min), different doses of PE (0.01 – 100 µM) were administered to the coronary vascular bed as a bolus injection. The vasoconstriction was recorded as a percentage increase in basal perfusion pressure in response to PE. In the pre-contracted coronary vascular bed with 10 μM PE, concentration-response curves to Ach (endothelium dependent vasodilator, 0.01 – 100 μM) and SNP (endothelium independent vasodilator, 0.001 – 0.1μM) were measured. The responses were expressed as percentage of relaxation of the PE-induced pre-contraction.

### 3.5. Drugs

Streptozotocin, acetylcholine chloride, phenylephrine hydrochloride, heparin sodium and sodium nitroprusside were obtained from Sigma (St. Louis, Mo). Sodium chloride, potassium chloride, magnesium sulfate, sodium hydrogen carbonate, potassium hydrogen orthophosphate, D-glucose and calcium chloride were obtained from Merck Laboratories and sodium pentothal from Rotex Medica, Germany.

### 3.6. Statistical Methods

Results were expressed as mean ± standard error (SEM) and comparisons between groups for each protocol were performed using repeated measurement ANOVA followed by LSD multiple comparison test or student t-test as appropriate using the SPSS software, Version 16.0, Chicago, IL, USA. A P-value of < 0.05 was considered significant.

## 4. Results

**Figure 1.**
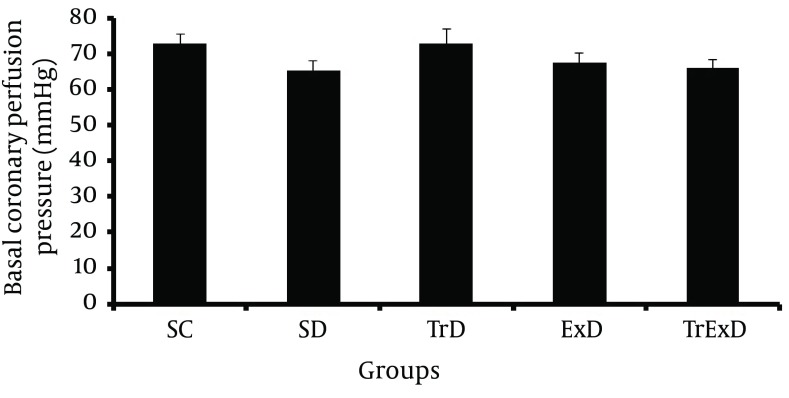
Basal coronary perfusion pressure (mean ± SEM, n = 7-8, CPP) of coronary vascular beds isolated from sedentary control (SC), sedentary diabetic (SD), trained diabetic (TrD), GSE treated sedentary diabetic (ExD) and GSE treated trained diabetic (TrExD) group. There was no significant difference between groups.

**Figure 2.**
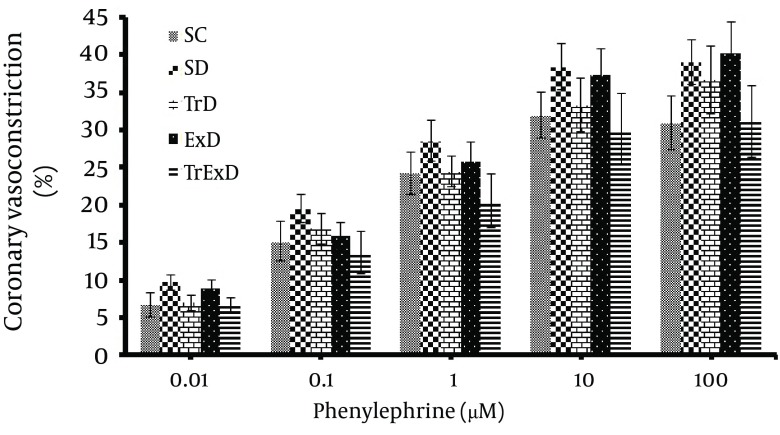
Vasoconstrictor responses (mean ± SEM, n = 8-10) to phenylephrine of coronary vascular beds isolated from sedentary control (SC), sedentary diabetic (SD), trained diabetic (TrD), GSE treated sedentary diabetic (ExD) and GSE treated trained diabetic (TrExD) group. There was no significant difference between groups.

Basal coronary perfusion pressure and vasoconstriction responses to phenylephrine in the isolated coronary vascular bed: Basal coronary perfusion pressure was not statistically different in all groups (SC: 72.7 ± 2.6, SD: 65.4 ± 2.6, TrD: 72.9 ± 3.8, ExD: 67.4 ± 2.8 and TrExD: 66.1 ± 2 mm Hg respectively) (Figure 1). Contractile response to phenylephrine (1 – 100 μM) increased dose dependently, but did not significantly change in different groups (Figure 2). 

**Figure 3.**
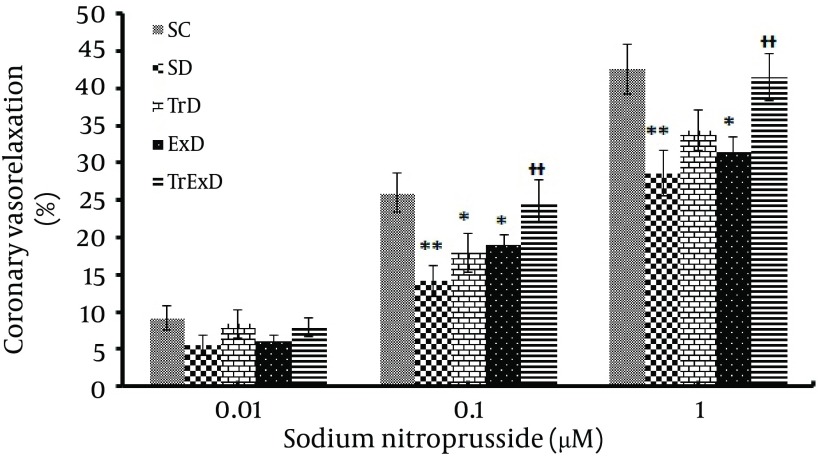
Vasodilator responses to sodium nitroprusside (Mean ± SEM, n = 7-8) in the phenylephrine (10 μM) pre-contracted coronary vascular beds isolated from sedentary control (SC), sedentary diabetic (SD), trained diabetic (TrD), GSE treated sedentary diabetic (ExD) and GSE treated trained diabetic (TrExD) groups. * P < 0.05, ** P < 0.01 indicates a significant difference from the sedentary diabetic group (tow way ANOVA followed by LSD).

**Figure 4.**
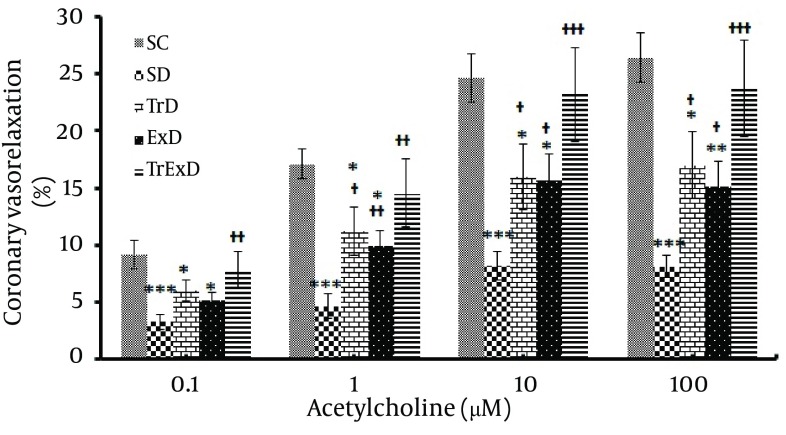
Ratio between maximum relaxation of different groups and control groups. Sedentary control (SC), sedentary diabetic (SD), trained diabetic (TrD), GSE treated sedentary diabetic (ExD) and GSE treated trained diabetic (TrExD) groups. * P < 0.05, ** P < 0.01 and *** P < 0.001 indicate a significant difference from the sedentary diabetic group and ‡ P < 0.05 and ‡‡ P < 0.01 demonstrate a significant difference from the control group (tow way ANOVA followed by LSD).

Vasorelaxation responses to sodium nitroprusside (coronary endothelium-independent relaxation): STZ significantly (P < 0.01) decreased the relaxation responses to SNP (0.01 – 1 µM) in the PE (10 μM) pre-contracted coronary vascular bed (i.e. % of maximum relaxation responses in control and diabetic rats were 42.6 ± 3.3 and 28.6 ± 3, respectively). Combined ET and GSE (P < 0.01) improved the relaxation response to SNP (41.5 ± 3.2) (Figure 3). % Maximum coronary independent dilation in SD, TrD, ExD, and TrExD groups was 67.3, 80.7, 74.1, and 97.5 respective of the control group (Figure 4). 

**Figure 5.**
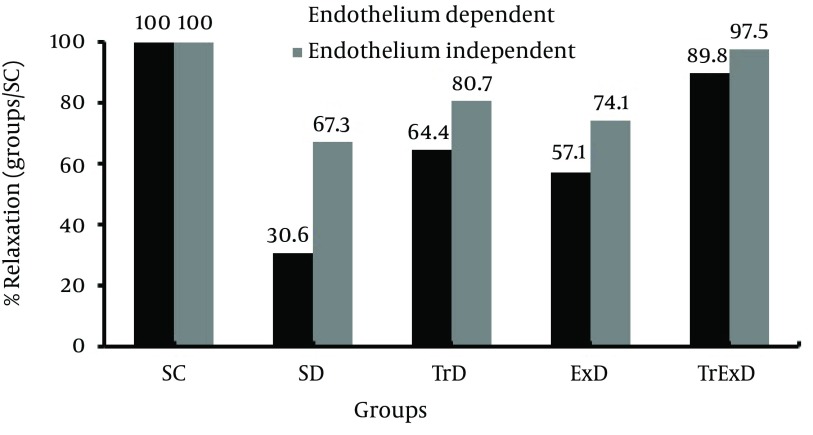
Vasodilator responses to acetylcholine (mean ± SEM, n = 7-8) in the phenylephrine (10 μM) pre-contracted coronary vascular beds isolated from sedentary control (SC), sedentary diabetic (SD), trained diabetic (TrD), GSE treated sedentary diabetic (ExD) and GSE treated trained diabetic (TrExD) groups.

Vasorelaxation responses to acetylcholine (Coronary endothelium-dependent relaxation); as shown in Figure 5, maximum coronary response to Ach in the control and diabetic groups pre-contracted with PE (10 µM) reached 26.46 ± 2.15 and 8.14 ± 1.27, respectively. This significant difference (P < 0.001) between SC and SD groups was partially (P < 0.05) improved by GSE (15.63 ± 2.3) or ET (17.03 ± 2.93) and perfectly (P < 0.001) restored by combined ET and GSE (23.76 ± 4.24). Percentage of Maximum coronary independent dilation in SD, TrD, ExD, and TrExD groups was 30.6, 64.4, 57.1, and 89.8, respective of that of the control group (Figure 4). 

## 5. Discussion

Our data showed STZ-induced diabetes and vasoconstriction response to PE of coronary vascular bed and that GSE with and without ET did not significantly change basal perfusion pressure. Endothelial independent relaxation to SNP and endothelial dependent relaxation to Ach was significantly reduced by STZ. Eight weeks of ET or GSE partially improved responses to Ach, but did not improve responses to SNP. Combined ET and GSE perfectly restored endothelial dependent and independent dysfunction induced by STZ.

Impairment of vascular function may contribute to the pathogenesis of vascular complications induced by diabetes (11, 17, 18). Some mechanisms of these impairments in diabetes may include factors that decrease endothelium derived relaxation factors such as reduced conversion of arginine to NO (19), increase in nitric oxide synthase (NOS) inhibitor (20), reduced NOS cofactor tetrahydrobiopterin bioavailability (21), and increase in endothelium derived relaxing factor (EDRF) destruction by oxidative stress (22), increase in endothelium-derived constricting factors (23, 24), endothelial cell apoptosis (25), and impairment of smooth muscle cell function (26).

In our study, DM impaired endothelium independent relaxation that is in accordance with other reports (27-29). Since relaxation to SNP (NO donor) was shown to be impaired, it is possible that vascular smooth muscle did not respond to NO. Reduction of endothelium dependent relaxation is well known and occurs in type 1 (12, 30, 31) and type 2 (32) diabetes. Relaxation induced by endothelium, reflects the function of both endothelial cells and smooth muscle cells. In our study, STZ-induced reduction of endothelial function was 69.4% whereas endothelium independent relaxation was only 32.7%. Therefore, endothelial impairment was 69.4% minus 32.7% equal to 36.7%.

In accordance to other reports (33, 34), ET partially improves endothelial dysfunction. The beneficial effects of ET might become available through improvement of hyperglycemia (our unpublished data) and insulin resistance that prevents impairment of endothelium dependent hyperpolarizing factor (EDHF) and EDRF, amelioration of diabetic-induced oxidative stress and by reduction of leukocyte adhesion (8), significant reduction in glycosylated hemoglobin (HbA1c), improvement of blood lipid levels, and decrement of blood pressure (7), improvement of arterial compliance (35), induction of angiogenesis and vasculature reactivity (36, 37), increment of NO production (37), increment of eNOS protein (38, 39), and increment of eNOS dimerization that increases coupling of the enzyme to facilitate production of NO (7).

It was shown that DM is associated with overproduction of oxidative stress (40-42). Oxidative stress may affect endothelial function through reduction of NO bioavailability or by serving as a contracting factor derived from endothelium (43) or by reduction of EDHF (44). EDHF hyperpolarizes the underlying smooth muscle via activation of K+ channels (45) or via gap junction (10) and thus relaxes the smooth muscles. It was reported that reactive oxygen species may induce gap junction dysfunction in diabetes (46).

GSE could improve diabetes-induced endothelial dysfunction. Vasorelaxation of GSE may be done via NO by phosphorylation of eNOS (47) and blockage of potential-dependent calcium channels and inhibition of calcium release (48). On the other hand, GSE is known as a powerful antioxidant (13). It was shown that antioxidants such as α-lipoic acid (9), Quercetin (49), xanthine oxidase inhibitor and allopurinol (10) have beneficial effects on both NO and EDHF mediated endothelium-dependent relaxation of diabetic states. Positive effects of vitamin E plus insulin in improving parameters of cardiac function and reduction of oxidative stress and apoptosis has been reported (50). Superoxide dismutase and tocopherol-acetate reduced perivascular fibrosis and significantly changed the contractile system in myocardium (51).

To our knowledge, this study is the first report showing the chronic beneficial effects of combined ET and GSE on coronary vascular function in diabetes. Complete improvement of endothelial function shows that this combination is more effective than ET or GSE alone. Since this study was not designed to find exact mechanisms of this positive combination, further experiments must be done to answer this question. Although the exact mechanisms of these actions are not clear, however the combination of the mechanisms mentioned above and other mechanisms may be considered.

Basal coronary perfusion pressure was not changed by diabetes, ET and GSE with and without ET. Since basal coronary perfusion pressure is affected by both nitric oxide (NO) and Prostaglandins (52), these mediators (NO and prostaglandins) are not affected by STZ or other interventions.

In our study, diabetes, ET, GSE and combined ET and GSE did not change the coronary vascular bed response to PE, which is in agreement with others (17, 53), although a reduction (10, 54) and increment (55) in this response have also been reported. Some factors that describe these discrepancies are attributed to the duration of diabetes and to the animal species and vascular bed studied (54).

In conclusion, the data indicated that ET combined with GSE administration had more significant effects than ET or GSE alone, may constitute convenient and inexpensive therapeutic approach to diabetic vascular complications.

## References

[1] Heller GV (2005). Evaluation of the patient with diabetes mellitus and suspected coronary artery disease.. Am J Med..

[2] Oltman CL, Davidson EP, Coppey LJ, Kleinschmidt TL, Lund DD, Adebara ET (2008). Vascular and neural dysfunction in Zucker diabetic fatty rats: a difficult condition to reverse.. Diabetes Obes Metab..

[3] Orimo M, Minamino T, Miyauchi H, Tateno K, Okada S, Moriya J (2009). Protective role of SIRT1 in diabetic vascular dysfunction.. Arterioscler Thromb Vasc Biol..

[4] Rodella LF, Vanella L, Peterson SJ, Drummond G, Rezzani R, Falck JR (2008). Heme oxygenase-derived carbon monoxide restores vascular function in type 1 diabetes.. Drug Metab Lett..

[5] Durante W, Sunahara FA, Sen AK (1989). Effect of diabetes on metabolic coronary dilatation in the rat.. Cardiovasc Res..

[6] Jansakul C, Hirunpan P (1999). Effects of exercise training on responsiveness of the mesenteric arterial bed to phenylephrine and KCl in male rats.. Br J Pharmacol..

[7] Grijalva J, Hicks S, Zhao X, Medikayala S, Kaminski PM, Wolin MS (2008). Exercise training enhanced myocardial endothelial nitric oxide synthase (eNOS) function in diabetic Goto-Kakizaki (GK) rats.. Cardiovasc Diabetol..

[8] Chakraphan D, Sridulyakul P, Thipakorn B, Bunnag S, Huxley VH, Patumraj S (2005). Attenuation of endothelial dysfunction by exercise training in STZ-induced diabetic rats.. Clin Hemorheol Microcirc..

[9] Cameron NE, Jack AM, Cotter MA (2001). Effect of alpha-lipoic acid on vascular responses and nociception in diabetic rats.. Free Radic Biol Med..

[10] Inkster ME, Cotter MA, Cameron NE (2007). Treatment with the xanthine oxidase inhibitor, allopurinol, improves nerve and vascular function in diabetic rats.. Eur J Pharmacol..

[11] Palmer AM, Thomas CR, Gopaul N, Dhir S, Anggard EE, Poston L (1998). Dietary antioxidant supplementation reduces lipid peroxidation but impairs vascular function in small mesenteric arteries of the streptozotocin-diabetic rat.. Diabetologia..

[12] Badavi M, Abedi HA, Sarkaki A, Dianat M (2011). Exercise Training and Grape Seed Extract Co-administration Improve Endothelial Dysfunction of Mesenteric Vascular Bed in STZ-induced Diabetic Rats.. Int J Pharmol..

[13] Shi J, Yu J, Pohorly JE, Kakuda Y (2003). Polyphenolics in grape seeds-biochemistry and functionality.. J Med Food..

[14] Okudan N, Sahin AS, Basal S (2011). The Effect of Supplementation of Grape Seed Proanthocyanidin Extract on Vascular Dysfunction in Experimental Diabetes.. J Med Food..

[15] Li BY, Cheng M, Gao HQ, Ma YB, Xu L, Li XH (2008). Back-regulation of six oxidative stress proteins with grape seed proanthocyanidin extracts in rat diabetic nephropathy.. J Cell Biochem..

[16] Badavi M, Mehrgerdi FZ, Sarkaki A, Naseri MK, Dianat M (2008). Effect of grape seed extract on lead induced hypertension and heart rate in rat.. Pak J Biol Sci..

[17] Fatehi-Hassanabad Z, Imen-Shahidi M, Fatehi M, Farrokhfall K, Parsaeei H (2006). The beneficial in vitro effects of lovastatin and chelerythrine on relaxatory response to acetylcholine in the perfused mesentric bed isolated from diabetic rats.. Eur J Pharmacol..

[18] Yousif MH, Oriowo MA, Cherian A, Adeagbo AS (2002). Histamine-induced vasodilatation in the perfused mesenteric arterial bed of diabetic rats.. Vascul Pharmacol..

[19] Avogaro A, Toffolo G, Kiwanuka E, de Kreutzenberg SV, Tessari P, Cobelli C (2003). L-arginine-nitric oxide kinetics in normal and type 2 diabetic subjects: a stable-labelled 15N arginine approach.. Diabetes..

[20] Lin KY, Ito A, Asagami T, Tsao PS, Adimoolam S, Kimoto M (2002). Impaired nitric oxide synthase pathway in diabetes mellitus: role of asymmetric dimethylarginine and dimethylarginine dimethylaminohydrolase.. Circulation..

[21] Bitar MS, Wahid S, Mustafa S, Al-Saleh E, Dhaunsi GS, Al-Mulla F (2005). Nitric oxide dynamics and endothelial dysfunction in type II model of genetic diabetes.. Eur J Pharmacol..

[22] Zou MH, Cohen R, Ullrich V (2004). Peroxynitrite and vascular endothelial dysfunction in diabetes mellitus.. Endothelium..

[23] Koltai MZ, Hadhazy P, Koszeghy A, Ballagi-Pordany G, Pogatsa G (1988). Prostaglandins and altered diabetic vasoregulation.. Biomed Biochim Acta Acad Med Wuhan..

[24] Shimizu K, Muramatsu M, Kakegawa Y (1993). Role of prostaglandin H2 as an endothelium-derived contracting factor in diabetic state.. Diabetes..

[25] Wu QD, Wang JH, Fennessy F, Redmond HP, Bouchier-Hayes D (1999). Taurine prevents high-glucose-induced human vascular endothelial cell apoptosis.. Am J Physiol..

[26] Witte K, Jacke K, Stahrenberg R (2002). Dysfunction of soluble guanylyl cyclase in aorta and kidney of Goto-Kakizaki rats: influence of age and diabetic state.. Nitric Oxide..

[27] Ajay M, Mustafa MR (2006). Effects of ascorbic acid on impaired vascular reactivity in aortas isolated from age-matched hypertensive and diabetic rats.. Vascul Pharmacol..

[28] Bender SB, Herrick EK, Lott ND, Klabunde RE (2007). Diet-induced obesity and diabetes reduce coronary responses to nitric oxide due to reduced bioavailability in isolated mouse hearts.. Diabetes Obes Metab..

[29] Vial G, Dubouchaud H, Couturier K, Lanson M, Leverve X, Demaison L (2008). Na+/H+ exchange inhibition with cariporide prevents alterations of coronary endothelial function in streptozotocin-induced diabetes.. Mol Cell Biochem..

[30] Baluchnejadmojarad T, Roghani M (2008). Chronic administration of genistein improves aortic reactivity of streptozotocin-diabetic rats: mode of action.. Vascul Pharmacol..

[31] Cinar MG, Ulker S, Alper G, Evinc A (2001). Effect of dietary vitamin E supplementation on vascular reactivity of thoracic aorta in streptozotocin-diabetic rats.. Pharmacology..

[32] Yang J, Park Y, Zhang H, Xu X, Laine GA, Dellsperger KC (2009). Feed-forward signaling of TNF-alpha and NF-kappaB via IKK-beta pathway contributes to insulin resistance and coronary arteriolar dysfunction in type 2 diabetic mice.. Am J Physiol Heart Circ Physiol..

[33] Desch S, Sonnabend M, Niebauer J, Sixt S, Sareban M, Eitel I (2010). Effects of physical exercise versus rosiglitazone on endothelial function in coronary artery disease patients with prediabetes.. Diabetes Obes Metab..

[34] Sixt S, Rastan A, Desch S, Sonnabend M, Schmidt A, Schuler G (2008). Exercise training but not rosiglitazone improves endothelial function in prediabetic patients with coronary disease.. Eur J Cardiovasc Prev Rehabil..

[35] Mourot L, Boussuges A, Campo P, Maunier S, Debussche X, Blanc P (2009). Cardiovascular rehabilitation increase arterial compliance in type 2 diabetic patients with coronary artery disease.. Diabetes Research and Clinical Practice..

[36] Delp MD (1995). Effects of exercise training on endothelium-dependent peripheral vascular responsiveness.. Med Sci Sports Exerc..

[37] Laughlin MH (1995). Endothelium-mediated control of coronary vascular tone after chronic exercise training.. Med Sci Sports Exerc..

[38] Johnson LR, Rush JW, Turk JR, Price EM, Laughlin MH (2001). Short-term exercise training increases ACh-induced relaxation and eNOS protein in porcine pulmonary arteries.. J Appl Physiol..

[39] Sessa WC, Pritchard K, Seyedi N, Wang J, Hintze TH (1994). Chronic exercise in dogs increases coronary vascular nitric oxide production and endothelial cell nitric oxide synthase gene expression.. Circ Res..

[40] Bubolz AH, Li H, Wu Q, Liu Y (2005). Enhanced oxidative stress impairs cAMP-mediated dilation by reducing Kv channel function in small coronary arteries of diabetic rats.. Am J Physiol Heart Circ Physiol..

[41] (2002). Role of hyperglycemia in nitrotyrosine postprandial generation.. Diabetes Care..

[42] Telci A, Cakatay U, Kayali R, Erdogan C, Orhan Y, Sivas A (2000). Oxidative protein damage in plasma of type 2 diabetic patients.. Horm Metab Res..

[43] Irving G. Joshua QZ, Jeff C.Falcone, Adrienne P.Bratcher, Walter E.Rodriguez, and Suresh C.Tyagi (2005). Mechanisms of Endothelial Dysfunction With Development of Type 1 Diabetes Mellitus: Role of Insulin and C-Peptide.. Journal of Cellular Biochemistry..

[44] Fukao M, Hattori Y, Kanno M, Sakuma I, Kitabatake A (1997). Alterations in endothelium-dependent hyperpolarization and relaxation in mesenteric arteries from streptozotocin-induced diabetic rats.. Br J Pharmacol..

[45] Quilley J, Fulton D, McGiff JC (1997). Hyperpolarizing factors.. Biochem Pharmacol..

[46] Oku H, Kodama T, Sakagami K, Puro GD (2001). Diabetes-induced disruption of gap junction pathways within the retinal microvasculature.. Invest. Opthalmol. Visual Sci..

[47] Edirisinghe I, Burton-Freeman B, Kappagoda T (2008). Mechanism of the endothelium-dependent relaxation evoked by a grape seed extract.. Clinical Science..

[48] Zhang TX, Niu CQ, Hu JM, Liu H, Jing HE (2008). [Vasorelaxational effects of procyanidins on rabbit aorta in vitro and decreasing arterial blood pressure in vivo].. Zhongguo Zhong Yao Za Zhi..

[49] Machha A, Achike FI, Mustafa AM, Mustafa MR (2007). Quercetin, a flavonoid antioxidant, modulates endothelium-derived nitric oxide bioavailability in diabetic rat aortas.. Nitric Oxide..

[50] Mohammad MD, Dimitri PM, Mohamed AH, Ismaeel MB, Olaa MT, Moshira AR (2008). Oxidative stress as a common mediator for apoptosis induced-cardiac damage in diabetic rats.. Open Cardiovasc Med J..

[51] Rosen P, Ballhausen T, Bloch W, Addicks K (1995). Endothelial relaxation is disturbed by oxidative stress in the diabetic rat heart: influence of tocopherol as antioxidant.. Diabetologia..

[52] Fatehi-Hassanabad Z, Furman BL, Parratt JR (1995). The effect of endotoxin on sympathetic responses in the rat isolated perfused mesenteric bed; involvement of nitric oxide and cyclo-oxygenase products.. Br J Pharmacol..

[53] Su J, Lucchesi PA, Gonzalez-Villalobos RA, Palen DI, Rezk BM, Suzuki Y (2008). Role of advanced glycation end products with oxidative stress in resistance artery dysfunction in type 2 diabetic mice.. Arterioscler Thromb Vasc Biol..

[54] Carvalho LAF, Coelho EB (2004). Effects of prostanoids on phenylephrine-induced contractions in the mesenteric vascular bed of rats with streptozotocin-induced diabetes mellitus.. Life Sci..

[55] Majithiya JB, Balaraman R (2006). Metformin reduces blood pressure and restores endothelial function in aorta of streptozotocin-induced diabetic rats.. Life Sci..

